# Systematic Review of Polygenic Gene–Environment Interaction in Tobacco, Alcohol, and Cannabis Use

**DOI:** 10.1007/s10519-019-09958-7

**Published:** 2019-05-20

**Authors:** Joëlle A. Pasman, Karin J. H. Verweij, Jacqueline M. Vink

**Affiliations:** 1 Behavioural Science Institute, Radboud University, Nijmegen, The Netherlands; 2 Amsterdam UMC, Amsterdam, The Netherlands

**Keywords:** Gene–environment interaction, Alcohol, Tobacco, Cannabis, Polygenic risk, Genetic risk

## Abstract

Studies testing the effect of single genetic variants on substance use have had modest success. This paper reviewed 39 studies using polygenic measures to test interaction with any type of environmental exposure (G×E) in alcohol, tobacco, and cannabis use. Studies using haplotype combinations, sum scores of candidate-gene risk alleles, and polygenic scores (PS) were included. Overall study quality was moderate, with lower ratings for the polygenic methods in the haplotype and candidate-gene score studies. Heterogeneity in investigated environmental exposures, genetic factors, and outcomes was substantial. Most studies (*N* = 30) reported at least one significant G×E interaction, but overall evidence was weak. The majority (*N* = 26) found results in line with differential susceptibility and diathesis-stress frameworks. Future studies should pay more attention to methodological and statistical rigor, and focus on replication efforts. Additional work is needed before firm conclusions can be drawn about the importance of G×E in the etiology of substance use.

**Electronic supplementary material:**

The online version of this article (10.1007/s10519-019-09958-7) contains supplementary material, which is available to authorized users.

## Introduction

The use of tobacco, alcohol, and cannabis continues to be widespread. Global smoking prevalence in individuals above age 15 is around 23% (World Health Organization 2016). On average, people drink a glass of alcohol per day, with higher estimates for America and Europe (World Health Organization 2014). Lifetime prevalence of cannabis use is 26% in the European Union and up to 44% in the United States (European Monitoring Centre for Drugs and Drug Addiction 2017; U.S. Department of Health and Human Services 2016). Risk factors on biological, social, and psychological level have been found to contribute to individual differences in substance use behaviors.

Genetic vulnerability is an important risk factor. Traditionally, this factor has been investigated using family and twin designs to determine how much variance in a trait is explained by genetic factors (Boomsma et al. 2002). Heritability estimates for substance use, abuse and dependence derived from these types of studies are moderate to high (about 30–75%; Ducci and Goldman 2012; Vink 2016).

Genetic molecular studies have tried to identify specific genetic variants underlying this heritability. In early studies the focus was on candidate-genes, selected based on their proposed biological function. In recent years, researchers have tried to identify genetic variants in a hypothesis-free manner in genome-wide association studies (GWASs), thereby focusing on single-nucleotide polymorphisms (SNPs). Although GWASs have had more success than candidate-gene studies, results are modest: only a handful of variants have been identified for substance use.

To increase power and because behavior is highly polygenic, studies have tried to test the effect of multiple genetic variants simultaneously. Some studies have used combinations of variants that are strongly related [i.e., are in high linkage disequilibrium (LD)] and are transmitted to offspring together in so-called LD blocks. The exact combination of alleles a person has on the variants in such a block is called a haplotype. It has been assumed that the effects of haplotypes are larger and thus easier to detect than those of single variants. Other studies have sought to increase power by combining several (unrelated) candidate-gene variants in a single sum score. A newer method uses summary statistics of GWASs to create weighted sums of the number of risk variants an individual carries, often called polygenic scores (PS). Research using haplotypes, candidate-gene sum scores, or PS has had some success in predicting substance use phenotypes. However, for all approaches, explained variance is still much smaller than expected based on the heritability estimates from twin research.

Possibly, explained variance can be increased by taking into account the interplay between genetic factors and the environment. In gene–environment interaction (G×E), the effect of a genetic factor depends on the presence of an environmental factor. The premise is that genetic factors underlie biological mechanisms (e.g., stress system responsivity) that make a person more or less vulnerable to environmental circumstances (Belsky and Pluess 2009). Indeed, twin research has shown that the extent to which genetic risk contributes to substance use can depend on environmental factors (Dick 2011). Early molecular genetic studies investigated G×E using single candidate-genes. For example, it was found that childhood maltreatment increased chances of early alcohol initiation more in carriers of the s-allele of the 5-HTTLPR polymorphism of the serotonin transporter gene than in non-carriers (Kaufman et al. 2007). This finding is one of many in line with the diathesis-stress model, stating that adverse environmental circumstances enhance the chance that genetic vulnerability comes to expression (Monroe and Simons 1991). Other G×E frameworks include the differential susceptibility model, posing that genetic predisposition might enhance the effect of adverse, but also of positive environmental factors (Belsky and Pluess 2009). Less commonly it has been predicted that more adverse outcomes arise when genetic plasticity is high and environmental risk is *either high or low,* as both might lead to high stress reactivity (Boyce and Ellis 2005).

Studies using single candidate-genes to test G×E in substance use and other complex phenotypes have yielded mixed findings (see e.g. Do and Maes 2016; Milaniak et al. 2015 for recent reviews). Non-replication and contradicting results seem the rule rather than the exception. The merits of the different theoretical G×E models remain unclear. Low powered study designs and publication bias are likely to have contributed to these mixed findings (Duncan and Keller 2011). To increase power, the logical next step has been to use polygenic rather than single-variant measures in G×E designs.

Whereas previous reviews focused on G×E with single (candidate) genes, this review presents a summary of G×E studies that used a polygenic measure, including haplotype-based measures, sum scores of risk alleles in candidate-genes, and PS based on SNPs identified in GWASs. We focused on (ab)use of and dependence on tobacco, alcohol, and cannabis, as these are the most frequently used substances, and most literature was available for these substances. No previous studies to our knowledge have attempted a review of G×E with polygenic measures, or developed a method to systematically review study quality. Because this field is relatively new, we included all G×E studies, regardless of the type of environmental exposure under investigation, ranging from cohort effects to childhood trauma. Based on our findings, methodological and theoretical recommendations for future research were formulated.

## Methods

For this review, PRISMA guidelines were used (Moher et al. 2009). The study method was preregistered in PROSPERO (CRD42017057478).

### Search strategy

Literature searches were conducted in Web of Science, PubMed and Google Scholar, and based on title and abstract potentially relevant articles were added. Only articles published in peer-reviewed journals were considered. Keywords included substance use, gene–environment interaction, and polygenic risk. The exact keyword combinations used can be found in supplemental Table SI. Reference lists were checked for additional articles. The last search was conducted February 1st, 2018.

### Study eligibility

Inclusion criteria were met if (a) the study included human subjects; (b) the outcome was some form of tobacco, alcohol, or cannabis use, or a combination thereof; (c) the study was an original research report; (d) the measure of genetic risk comprised a combination of multiple risk variants (i.e., no single variant designs); and (e) an interaction with an environmental variable was tested statistically. Criterion d allowed for studies that looked at multiple variants within one gene. Although this kind of study does not meet the strict definition of ‘polygenic’, it might be more powerful than studies looking at only one variant (Oroszi et al. 2009). Earlier reviews of candidate-gene studies have not explicitly investigated the merits of this method. Criterion e allowed for any demographic/environmental factor that has been investigated in this context, including for example birth cohort or something as specific as roommate’s alcohol use levels in high school.

### Assessment of study quality

For each study, quality characteristics were assessed. Important hallmarks included study design, sample size, power (sample sizes necessary to achieve different levels of power are described in Supplementary Table SII), the method for controlling for confounders (sex, age, population stratification/ethnicity, gene–environment correlation), and phenotype measurement. The quality of the operationalization of the polygenic measure was assessed separately for the haplotype, candidate-gene score, and PS studies.

As no scale exists for assessing the characteristics of this specific type of study, study quality was visualized using symbols (−, +−, +). Symbol allocations for study characteristics are summarized in Table 1. Although literature was consulted for handholds (Table 1), quality cut-offs had to be chosen without objective reference points. Assessment of study quality was done in duplicate (JP, KV); any disagreement was solved through discussion with a third assessor (JV).

**Table 1:** Symbol allocation for quality characteristics of the G×E studies

Method	Characteristic	−	−+	+	Not applicable
All	Study type	Correlational	Case control	Randomized	
	Sample size	< 1000	1000–2500	> 2500	
	Power calculation	No	–	Yes	
	Control for age and sex^a^	None	Descriptive	Statistical	Homogenous sample/age as predictor or outcome
	Control for ethnicity^b^	None	Descriptive	Statistical	Homogenous sample
	Control for rGE^c^	None	Descriptive	Statistical	Interventions/cohort effects
	Phenotype measures	Self-developed short survey	Validated survey/interview	Biological/combined measures	Interventions/cohort effects
Haplotype	# of blocks^d^	1–4	–	> 4	
	# of genes^d^	1–3	–	> 3	
	# of variants^d^	< 5	5–10	> 10	
	Rationale for risk haplotype^e^	Debatable	–	Solid	
Candidate	# of genes^d^	1–3	–	> 3	
	# of variants^d^	< 5	5–10	> 10	
	Rationale for risk allele	Debatable	–	Solid	
Polygenic score (PS)	Based on	Overlapping sample GWAS	–	Independent GWAS	
	Discovery sample size	< 10,000	10,000–25,000	> 25,000	
	*p* value threshold^f^	*p *< .0001	–	*p *≥ .0001	
	Correspondence phenotypes^g^	Weak	Moderate	Strong	

^a^Genetic associations may vary in different age and sex groups (Kendler et al. 2008; The Wellcome Trust Case Control Consortium 2007)

^b^Population stratification resulting from ancestry differences can distort genetic association results (Price et al. 2006); statistical control using principal component analysis is preferable to control for these effects

^c^In gene-environment correlation (rGE) genetic make-up influences to what environment an individual is exposed (only possible in non-randomized studies). These effects can muddle G×E findings (Rathouz et al. 2008a, b)

^d^Inclusion of more genetic factors in the aggregate predictor was considered better. Cut-offs were based on commonly chosen numbers of variants for these studies

^e^The rationale for defining which haplotype or allele was the active (risk/protective) allele was deemed less strong when it was based on the results of the main analyses in the same sample, rather than on theory or results from independent samples

^f^This threshold most commonly concerns the *p* value for the association between the SNPs and the phenotype in the original GWAS. The lower this value, the fewer SNPs are included in the PS. We considered PS including only a few SNPs as less strong than PS including more SNPs, although the exact optimal threshold depends on several other study characteristics (Chatterjee et al. 2013; Dudbridge 2013)

^g^The more similar the outcome variable is to the original GWAS phenotype on which the PS was based, the better the predictive value (Wray et al. 2014)

### Data extraction and evaluation of results

The studies were categorized according to (a) the measure of genetic risk (haplotype, candidate-gene score, or PS), and (b) the nature of the environmental exposure (intervention or other, e.g., traumatic experiences). Further categorization could not be realized due to heterogeneity in environmental factors, outcomes, and study designs. No meta-analysis nor formal publication bias assessment could be attempted because of study heterogeneity, inconsistent statistical reporting, and absence of report of (standardized) effect sizes.

As most studies did report *p* values for the G×E analysis, a *p* curve analysis could be conducted (Simonsohn et al. 2014) to give an indication of the strength of the evidence and of the probability that *p* hacking occurred in the included studies. The assumption of the *p* curve method is that if the investigated effect is real, there should be more small *p* values than large *p* values reported in the literature. If there are more large than small *p* values, this might be seen as evidence for *p* hacking or selective reporting; it is more likely that investigators have been conducting tests until they reached a *p* value just below .05. Supplementary Table SIII summarizes what G×E test statistics were selected from each study. The analysis was conducted twice, once using all the reported G×E *p* values in each study, and once using only the first reported *p* value in each study. If a study only reported that the *p* value was smaller than some threshold (e.g., *p *< .05, *p *< .001), we included this threshold minus one decimal value (e.g., *p *= .049, *p *= .00099) as the estimated *p* value in the analysis.

## Results

### Selection

**Fig. 1:**
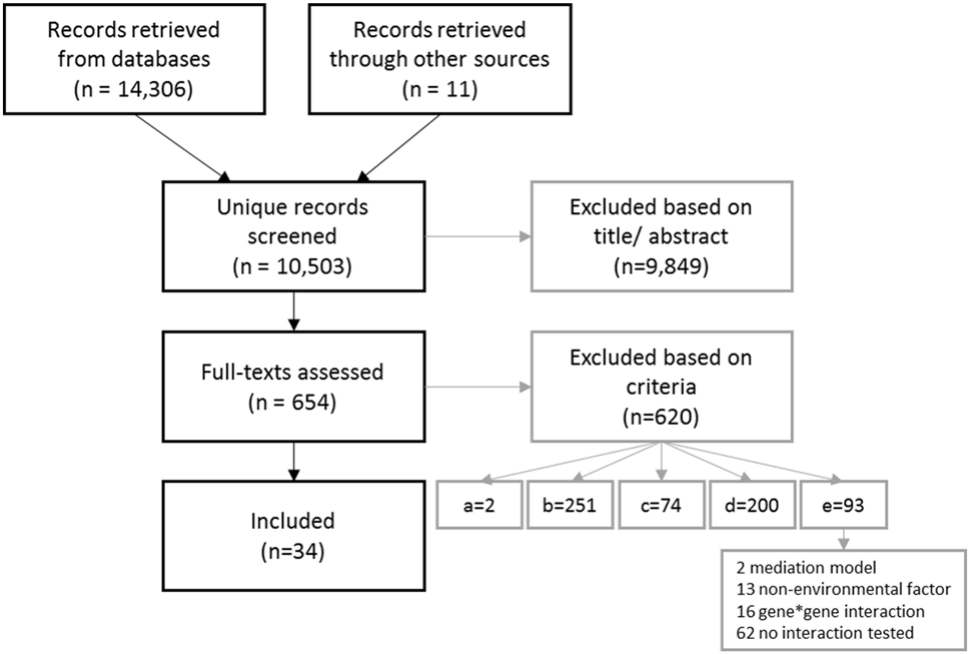
Flow-chart of study selection for inclusion in the review. Exclusion criteria: **a** non-human subjects; **b** no substance use outcome; **c** no original research; **d** no polygenic risk predictor; **e** no statistical test of interaction with environmental variable

The study selection process is summarized in the flow chart in Fig. 1. In total, 34 articles describing 39 studies were left for inclusion in the systematic review. These studies described results from 27 independent samples.

### Study description

In Tables 2, 3, and 4 key features and G×E findings are summarized separately for studies using (a) haplotype, (b) candidate-gene score, and (c) PS measures. Symbols are used to annotate what studies used data from overlapping samples.

**Table 2:**
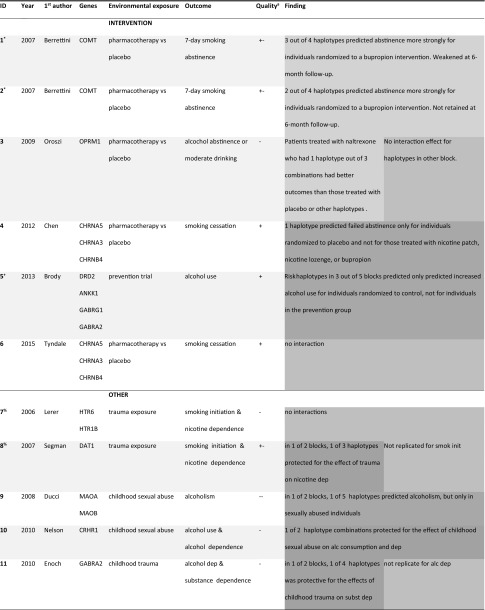

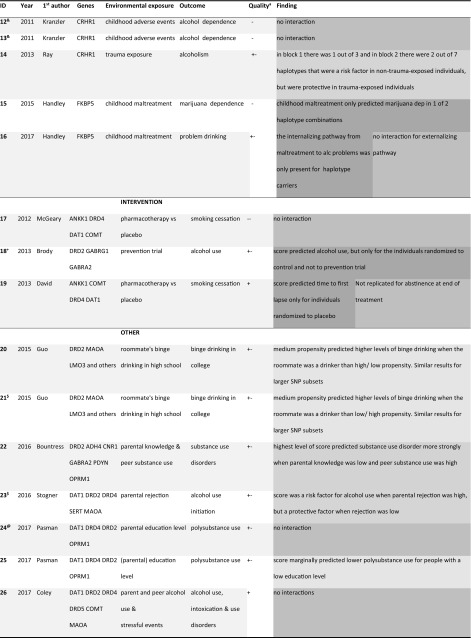

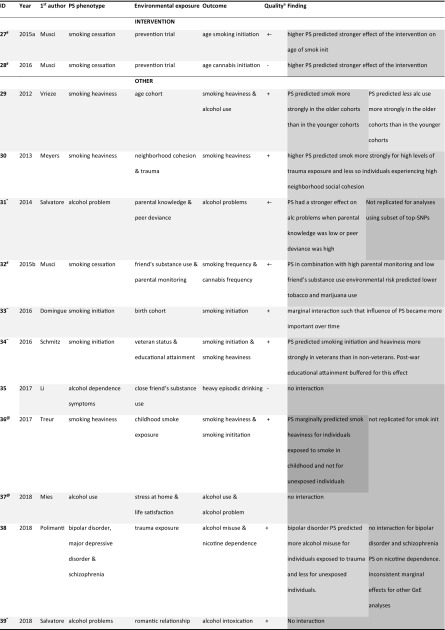
Summary of G×E studies using haplotypes as a measure for polygenic risk (G)

Top rows for studies testing intervention/prevention as environmental exposure (E). Only first author of the respective papers is mentioned

Green = reinforcing, dark green = reinforcing such that G only has effect in one E, blue = E has only effect for one level of G, orange = G’s effect is reversed by E, gray = no evidence for G×E

*^,+,%,&,$,@,#^Studies denoted with the same symbol used data from identical or overlapping samples

^a^Quality ratings based on characteristics from Supplementary Tables SVa-SVc

Sample characteristics for each study are given in Supplementary Table SIV. Samples consisting of only European descent individuals were overrepresented (51%). Studies included clinical (*N* = 8), clinically ascertained (*N* = 5), and general populations (*N* = 26). Eleven studies included family-related individuals. Studies comprised various age ranges starting from adolescence, with 19 studies specifically focusing on adolescents or young adults and two on older adults. There was an approximately equal representation of female and male subjects within the studies. Sample sizes ranged from *N* = 81 to *N* = 11,423, with an average of *N* = 1865.

Fifteen studies used some form of correlational design (7 longitudinal), 12 were case–control studies, 11 were RCTs and 1 was a randomized longitudinal design. Twenty studies included alcohol and 16 included tobacco outcomes (among others), 4 focused on combined phenotypes (e.g., substance use disorder), and only 3 included cannabis outcomes. There were 11 intervention studies, 12 studies that included measures of trauma-like experiences, and 16 that focused exclusively on typical environmental exposures in for example the family or peer context. Most haplotype studies focused on interventions or psychological trauma as environmental exposures, whereas the candidate-gene score and PS studies more often focused on common environmental factors.

### Quality

General quality characteristics per study type are summarized in Table 3. Quality of the implementation of the polygenic method is summarized in Table 4. Full details on quality characteristics per study are given in Supplementary Tables SVa–SVc.

**Table 3:** Summary of general study quality per category, expressed in percentage of studies that met a criterion as specified and assigned with a −, +−, or + in Table 1

		Haplotype	Candidate-gene score	Polygenic score (PS)
Design	% correlational	0%	60.0%	69.2%
	% case control	62.5%	0%	15.4%
	% randomized	37.5%	40.0%	15.4%
	Rating	+	+−	−
Sample size	M (SD)	*N* = 771 (658)	*N* = 2141 (3159)	*N* = 3001 (3439)
	% with *N* ≥ 1000	31.3%	60.0%	53.8%
	Rating	−	−	+−
Power	% reported	37.5%	30%	38.5%
	Rating	−	−	−
Control for confounders	% age statistically controlled^a^	60.0%	88.9%	100%
	% sex statistically controlled^a^	70.0%	100%	100%
	% ethnicity statistically controlled^a^	42.8%	83.3%	100%
	% rGE reported^b^	40.0%	50.0%	80.0%
	% rGE statistically controlled^b^	0%	16.7%	10%
	Rating	−	+−	+
Phenotype measures^c^	% self-developed short survey	12.5%	60.0%	72.7%
	% validated survey/interview	62.5%	30.0%	27.2%
	Biological/combined measures	25%	10.0%	0%
	Rating	+	−	−

For details per study, see Supplementary Tables SVa–Vc

^a^When sample was sufficiently homogeneous, the study was not considered for calculating this percentage

^b^When rGE could not be an issue (in the case of cohort effects or intervention studies) the study was not considered for calculating this percentage

^c^Only percentages for the outcome measures are mentioned here. For details on environmental measures, see Supplementary Tables SVa–Vc

**Table 4:**
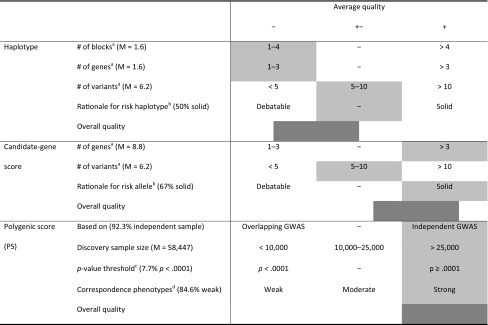
Summary of quality of the implementation of the polygenic method

Averages or counts are given per criterion. Shading indicates that most studies (or the study average) fell into this quality category, with the darker shading indicating the average quality category per study type. For details per study, refer to Supplementary Tables SVa–SVc

^a^Inclusion of more genetic factors in the aggregate predictor was considered better. Cut-offs were based on commonly chosen numbers of variants for these studies

^b^The rationale for defining which haplotype or allele was the risk/protective allele was deemed less strong when it was based on the results of the main analyses in the same sample, rather than on theory or results from independent samples

^c^This threshold most commonly concerns the *p* value for the association between the SNPs and the phenotype in the original GWAS. The lower this value, the fewer SNPs are included in the PS. We considered PS including only a few SNPs as less strong than PS including more SNPs, although the exact optimal threshold depends on several other study characteristics (Chatterjee et al. 2013; Dudbridge 2013)

^d^The more similar the outcome variable is to the original GWAS phenotype on which the PS was based, the better the predictive value (Wray et al. 2014)

### Haplotype method

Haplotype studies (*N* = 16) were on average published 7 years before the date of inclusion in this review. They used a strong experimental design (i.e., RCT or case control) more often than both other study types. Sample sizes were quite low given the expected small effects, with an average of ${\hat{\text{N}}}$ = 771 and almost half of the studies using a sample of less than *N* = 500 individuals. In many cases, exact sample sizes were only reported for the main effects analysis and not for the G×E analysis. Power calculations were not reported in 10 out of 16 studies.

Only three studies controlled statistically for both age and sex, although other studies often reported that outcomes and predictors did not vary for different sexes or age groups (‘descriptive’ control). In a few cases, statistical control was unnecessary as the sample was sufficiently homogeneous (e.g., all female or all within the same 2-year age range). Control for ethnicity was absent or rudimentary in all but one study (16), for example consisting of self-reported ‘white/non-white’ racial background. In nine studies the sample was reasonably ethnically homogeneous. In non-randomized studies (*N* = 10) gene–environment correlation (rGE) might confound the G×E interaction results. Only four of the ten studies reported on rGE and no studies controlled for these effects.

The quality of the application of the haplotype method was limited. Almost all studies tested haplotypes in one or two LD blocks, with the number of tested variants ranging between 2 and 18. Many studies looked at 1 or 2 blocks in a single gene or a few genes in high LD, so that they are hardly more ‘polygenic’ than single candidate-gene studies. Most studies did not formulate a literature-based directional prediction. In many cases, only haplotypes that showed a main effect on the outcome were included in the G×E analysis.

For severe outcomes (e.g., clinical diagnosis) or environmental exposures (e.g., traumatic experiences) interviews were used as a measurement instrument. In the smoking cessation trials, biological measures were employed to validate self-reported abstinence. Other outcomes and exposures were mostly measured using validated questionnaires or more crudely using short questionnaires that were developed for the purpose of this or an earlier study.

Most haplotypes investigated were located in genes involved in (dopamine-related) reward and inhibition processes in the brain (e.g. *COMT, ANKK1, DRD2, DAT1, OPRM1, HTR6, HTR1B,* and *GABA*- and *MAO*-related genes). Other candidate-genes included *CRHR1* and *FKBP5*, related to the stress system. Genes such as the nicotine metabolism, cannabinoid receptor, or the alcohol dehydrogenase genes seem equally suitable candidates, but received much less research attention.

Summarizing, weaknesses of the haplotype studies included small sample sizes, low statistical control, and limitations in the implementation of the polygenic method. Strengths included the use of strong designs and phenotypical measures.

### Candidate–gene method

Studies using candidate-gene scores were on average 3 years old at the time of inclusion in this review. The study designs were somewhat less strong than those used in the haplotype studies, with four out of ten using some randomization procedure. Average sample size was larger than for the haplotype studies (${\hat{\text{N}}}$ = 2141), although for 2 studies exact sample size for the G×E analyses were not reported and the average was boosted by one study with *N* = 11,423. Three out of ten studies reported power calculations.

Control for confounders was more stringent than in the haplotype studies, with 8 studies exerting statistical control for both age and sex. Five studies used some control for genetic ancestry and another four used a relatively homogenous sample. One of the seven studies that did not use a randomization procedure statistically controlled for rGE effects and three studies reported on them.

All but one candidate-gene score study used an unweighted sum score of the number of risk alleles as a predictor. Sum scores were based on risk alleles in on average seven variants. Almost all variants were located in previously investigated candidate-genes related to dopamine-signaling. The rationale for selecting the risk allele was debatable in three cases. Many studies did omitted a description of conflicting literature on the risk allele of the candidate-gene. Outcome and environmental exposure measures were generally of lower quality than those in the haplotype studies and mostly comprised self-developed short questionnaires.

Candidate-gene studies scored slightly better than the haplotype studies on sample sizes, control for confounding, and the implementation of the polygenic method.

### Polygenic score method

Studies using PS were the newest, on average 2 years old at the time of inclusion. Study designs were more often simple correlational designs. Sample size was ${\hat{\text{N}}}$ = 3001 on average, with only two samples smaller than *N* = 500. Five out of 13 studies reported power calculations.

Control for confounding was most rigorous in this type of studies, with seven studies controlling statistically for both age and sex, and the rest using some procedure rendering statistical control less necessary. In PS studies it is possible to control statistically for ethnicity using ancestry-informative principal components. Eight of the ten studies used this procedure, with an additional two using a more rudimentary approximation of this method and the final three using a reasonably homogenous sample. Of the ten studies where rGE could have played a role, one exerted statistical control for rGE and eight described the effects without controlling for them.

Half the studies constructed the PS based on results from larger GWASs (average discovery ${\hat{\text{N}}}$ = 58,447, range *N* = 31,266–74,053), while the others based it on results from GWASs with limited sample size (${\hat{\text{N}}}$ = 3140). One of the latter calculated the PS based on a GWAS in a sample that was genetically related to the target sample, which could have biased results (35). The similarity between the study outcome and the source GWAS phenotype was in most cases reasonably high. The specific score calculation method differed somewhat across studies, with three preselecting a subset of SNPs to include in the score. Most studies used the PLINK program to calculate the PS, using pruning or clumping to remove variants that were in high LD. One study used LDpred to calculate PS while accounting for this LD (36). The *p* value thresholds for including SNPs in the PS varied widely from *p *< 1 × 10^−8^ (resulting in a score of only two variants) to *p *= 1 (retaining all SNPs in the score), and five studies tested multiple PS with different thresholds.

Overall, the phenotype measures were of limited quality, with most studies using short self-developed questionnaires.

Overall, the PS studies scored higher than the other study types on sample size, control for confounding, and the implementation of the polygenic method, but similar or worse on study design and phenotypical measures.

In general, study quality appeared somewhat higher for studies that found a significant G×E result than for studies that did not. This might have been driven by the higher quality of the PS studies, that could also have yielded more significant findings because of higher power. Study quality did not seem to influence what kind of G×E pattern was found.

## Main results

### *p* curve analysis

**Fig. 2:**
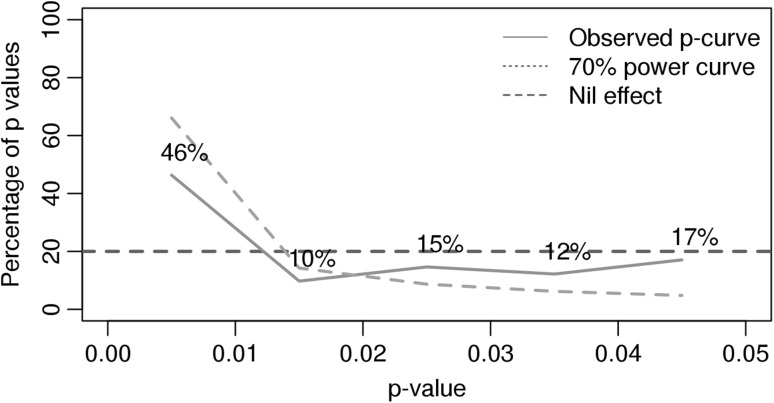
Percentage (*y* axis) of reported *p* values in the studies that fell in the range specified on the *x* axis (*p* curve), against the percentage that would be expected under the null hypothesis (nil effect) and under the alternative hypothesis given a power level of 70% (70% power curve)

To get an indication of the overall evidence for G×E in substance use, a *p* curve analysis was conducted. The analysis was based on 82 *p* values (34 significant) derived from 28 studies (see Supplementary Table SIII). The other 11 studies did not report *p* values for the G×E term specifically (or for the simple effects in the case of cross-over interactions) or statistics by which these could be calculated. As can be seen in Fig. 2, there were more small *p* values than expected under the null hypothesis. The *p* curve is flatter than expected if studies had at least 70% power, such that there were not many more small *p* values than medium to large *p* values. This indicates that there was evidential value, but this was not very strong. Moreover, if only the first *p* value reported in each study was taken into account, results deteriorated, indicating that they were driven by a few studies that reported many small *p* values (data not shown). Results did not change if non-exact *p* values (e.g., *p *< .05) were excluded from analysis (results not shown). There was no clear evidence for *p* hacking, which would be indicated by a substantially higher proportion of *p* values just below 0.05.

### G×E patterns

**Fig. 3:**
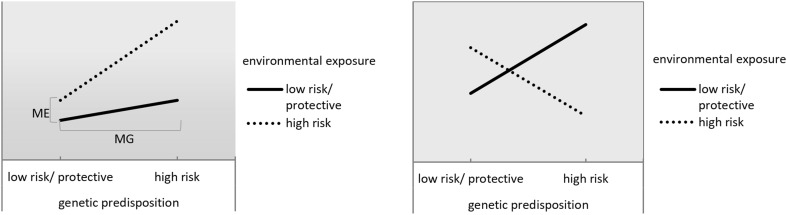
General pattern of G×E. **a** Genetic factors and environmental factors reinforce each other (green–blue shades in Tables 2, 3, and 4, *N* = 21). ME represents the main effect of environmental exposure, MG that of the genetic factor. **b** The effect of a genetic factor is reversed as a function of an environmental factor (or vice versa; orange findings in Tables 2, 3, and 4, *N* = 2)

The G×E findings from each of the 39 studies were summarized in the last column of Tables 2, 3, and 4. Thirty studies reported at least 1 significant G×E finding and 9 did not. Twenty-five of the significant G×E findings followed a pattern as depicted in Fig. 3a (22 studies) or b (3 studies). The pattern in panel a indicates that environmental risk enhances the effect of genetic risk, further increasing the chance of unfavorable outcomes. Or, likewise, a protective genetic predisposition might enhance the effects of a positive environment or counteract the effects of an adverse environment. Thus, in these cases, genetic and environmental factors *reinforce* each other’s effects. In panel b the pattern is similar, only now a genetic factor that is a risk factor in one situation, is protective in the other situation, or likewise, an environmental exposure that is a risk factor for individuals with a certain genetic make-up is a protective factor for individuals with a different genetic make-up. Thus, genetic and environmental factors *reverse* each other’s effects (cross-over interaction).

The colors in Tables 2, 3, and 4 correspond to specific patterns of results as summarized in Fig. 3. The light green color indicates that the study interpreted the G×E effects such that the genetic and environmental factors reinforce each other (study 1–3, 21, 26–31, 33). For example, in study 22 a genetic risk factor (associated with substance use) enhances the effect of an adverse environmental factor (high peer substance use), yielding a negative outcome (substance use disorder).

Studies marked in darker green are similar, but did not find (or report) a main effect of environment (‘ME’ in Fig. 3a) or a main effect of the genetic factor (‘M’ in Fig. 3a; study 4–5, 9, 15–16, 18–19, 36). The interpretation in these cases is that genetic risk *only* has an adverse effect in an adverse situation (study 9, 15, 16, 36) or when there is no intervention to counteract it (study 4, 5, 18, and 19). Likewise, the blue studies find that environmental risk only has an adverse effect in the absence of a protective genetic factor. The 3 studies finding this pattern are all haplotype studies where a specific combination of alleles protects for the effect of psychological trauma (studies 8, 10, 11).

Studies marked in orange (14, 23, and 38) showed that a genetic risk factor becomes a protective factor depending on the environment as depicted in Fig. 3b. For example, in study 14, certain haplotype combinations were risk factors for alcoholism in controls, but protective factors in traumatized individuals.

The yellow studies find patterns that correspond with neither Fig. 3a nor b. Study 20 and 21 find that *medium* levels of genetic risk predict adverse outcomes for high environmental risk. Study 25 reports that high genetic risk predicts *favorable* outcomes for an adverse environmental characteristic (low parental education). Study 29 found a PS for smoking heaviness to be related to less alcohol use for older cohorts, whereas it was related to more smoking in this group. Another cohort study (33) reported that genetic risk marginally predicted adverse outcomes more strongly for young cohorts, even though being in such a cohort is generally viewed as protective for substance use.

### Patterns substances, genes, and environments

Patterns of G×E did not seem to differ depending on the substance under investigation. Fourteen of the 20 studies including alcohol outcomes, and 13 of the 16 studies including smoking outcomes found at least one significant G×E effect.

Results did not seem to differ depending on the kind of variants investigated. For example, studies looking at dopamine-related genes were not more likely to find significant G×E patterns than studies focusing on other candidate-genes. However, it was difficult to compare findings across gene-groups, as most studies used aggregates of genes from different groups.

Intervention studies and studies looking at trauma exposure seemed more likely to yield patterns corresponding to Fig. 3a or b, but not more likely to show significant results. The 16 studies focusing on common environmental exposures yielded more diverse patterns. For example, all 5 yellow outcomes fell in this category, investigating birth cohort, peer substance use, and parental education level.

## Discussion

The aim of this review was to provide an overview of all available studies (*N* = 39) using measures of polygenic risk (haplotypes, candidate-gene scores, and polygenic scores) to investigate gene–environment interaction in substance use. There was some support for the existence of G×E in substance use, but the evidential value was weak.

### Theoretical interpretation

Most G×E results followed the pattern as depicted in Fig. 3a. These patterns nicely fit in the diathesis-stress framework (Monroe and Simons 1991), stating that individuals who are at risk genetically show higher levels of some adverse outcome when they are exposed to a risk environment. Although not stated in the original model, the same seems to apply for individuals who have a protective genetic predisposition in that they have more positive outcomes in beneficial environments. It is important to point out that this would fit equally well in the differential susceptibility framework (Belsky and Pluess 2009), but it is rarely found (or reported) within studies that the same genetic factor has a positive effect in one situation and a negative effect in the other. Only three studies report such an effect (14, 23, and 38), providing direct evidence for a ‘genetic plasticity factor’ yielding differential susceptibility.

Many studies did not provide a strong theoretical framework for predicting one G×E pattern rather than another. It seems that a fitting theoretical explanation can be found regardless of the pattern that was discovered. For example, findings that environmental factors have a stronger effect at *medium* levels of genetic risk have been explained lending from a ‘social push’ model framework, stating that a risk factor is overruled at particularly low or high levels of another risk factor (Guo et al. 2015). Researchers may be tempted to place their findings in a theoretical framework a posteriori, rather than formulating hypotheses beforehand. Pre-registering hypotheses might be a good way to overcome these caveats.

As heterogeneity in outcomes, genetic predictors, and environmental factors was substantial, it was difficult to discern patterns in the results. These did not appear to depend on gene group, environmental factor, or substance investigated. However, patterns were hard to discern, as there were many different (combinations of) factors investigated.

### Limitations of included studies

Study quality differed within and between genetic risk assessment methods, and was often limited. For all study types, power calculation was mostly omitted, and many studies are likely to have been underpowered (see Table SII). Interaction effects usually require more power to be detected than main effects, and given that main effects of genetic predictors are often small this is especially relevant in G×E studies (Duncan and Keller 2011). For example, if the effect size of a G×E effect would be R^2^ = 0.5%, in order to achieve 80% power sample size would need to be *N* = 2185 (assuming 3 predictors and α = .05), and 31 out of 39 studies had smaller sample size than that (see Table SII). To put that in perspective, main effects of top SNPs in GWASs are often around 0.25%, and all measured SNPs together typically explain around 10% or less of the variance in substance use phenotypes (So et al. 2011). Also, control for gene–environment correlation was limited, and where it was tested, the test often entailed a simple correlation without controlling for the effects of covariates, or for interaction effects between the genetic predictor and covariates (Keller, 2014). This is problematic, as many environmental factors (such as parenting behaviors) are in fact influenced by genes themselves (Krapohl et al. 2017), and this covariation would decrease chances of detecting G×E and impede its interpretation (Rathouz et al. 2008a, b). Over all study types, heterogeneous designs, lack of replication studies, and inconsistent statistical reporting made assessment of publication bias impossible. In candidate-gene G×E studies such bias to underreporting negative results has been demonstrated (Duncan and Keller 2011). As the number of *p* values just below the significance threshold was not higher than the number of small *p* values, we concluded that *p* hacking did not seem to be an issue.

The haplotype method was limited as a measure of polygenic risk because many studies looked at a few variants in one gene, which is strictly speaking not ‘polygenic’ (but ‘polyvariant’) and will not capture much variation. The investigated genes were mostly plausible candidates for substance use because of their biological function. The benefits of this method compared to the traditional single candidate-gene method are modest. This might be reflected in the results, as more haplotype than other studies did not find a G×E interaction or found results that are difficult to interpret (i.e., did not follow a pattern as depicted in Fig. 3).

Studies using candidate-gene score methods appeared of somewhat better quality than the haplotype studies. Sample sizes and the number of investigated variants still seem (too) low to detect small effects (Luan et al. 2001). A more fundamental drawback of the candidate-gene method in general is that the selection of variants and risk alleles by definition has to rely on a limited body of knowledge, that might or might not include information on the causally most important genetic variants (Zhu and Zhao 2007). As an example, previous research has shown that candidate-genes for schizophrenia did not predict schizophrenia better than candidate-genes for an unrelated phenotype (diabetes; Johnson et al. 2017). Indeed, few of the proposed candidates in haplotype or candidate-gene score studies have actually been identified in hypothesis-free GWASs (e.g., Liu et al. 2019; Pasman et al. 2018; Walters et al. 2018). This might have added to the finding that studies using these methods more often yielded unexpected patterns.

Technical advances and decreasing costs have made it possible to consider the whole genome for risk prediction, and studies using such PS seem to become more popular than those using haplotype and candidate-gene methods. PS studies yielded the highest quality ratings, with sample sizes more adequate to capture small effects, although study design and phenotype measurements were less strong. It is important to note that PS studies are not necessarily appropriate for testing differential vulnerability hypotheses, as SNPs that operate through that mechanism would not necessarily have large main effects likely to be detected in a GWAS (Fox and Beevers 2016). It is an interesting possibility that different variants are important for interaction effects than for main effects, and this might contribute to the fact that G×E studies show disappointing results in comparison with GWASs. Furthermore, even the qualitatively better studies reviewed here show only small effects.

## Recommendations for future studies

**Fig. 4:**
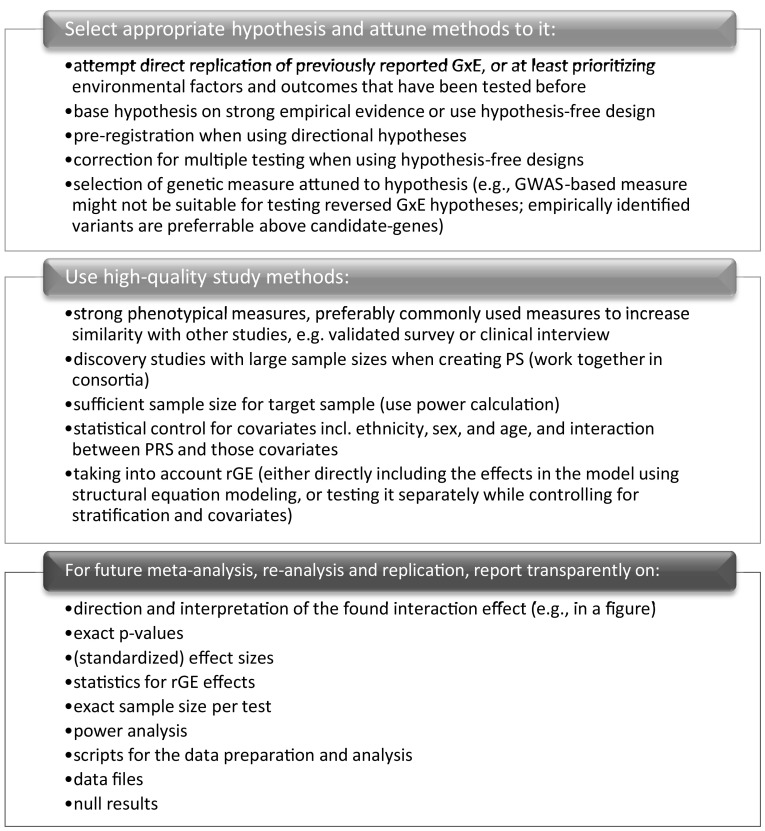
Road map for future studies with recommended steps for improving the stance of the substance use G×E literature

Following from the limitations of the included studies, important recommendations for future research can be made. A roadmap for future research is summarized in Fig. 4. First, more attention should be given to hypothesis selection. Although addiction research would be advanced by a further expansion of the scope of research, direct replication attempts might be even more important at this stage (Duncan and Keller 2011). Replication and original studies alike should focus on formulating and pre-registering sharp predictions and give attention to the exact direction of the G×E effects (Belsky et al. 2013; Munafò et al. 2017).

Second, high quality study methods should be used. Despite having limitations, the PS studies yielded more consistent results than the other two study types. GWASs with substantial sample sizes for alcohol (*N* ≈ 941,000) and tobacco use (*N* ≈ 1,232,000; Liu et al. 2019), and cannabis use (Pasman et al., 2018; *N* ≈ 184,000) are increasingly available, enhancing the predictive power of PS. It is interesting to note that studies are emerging testing SNP by environment interactions in GWASs, making it possible to explore G×E in a hypothesis-free manner. This would circumvent the difficulty that SNPs captured in GWASs do not necessarily measure differential susceptibility. For instance, Polimanti et al. (2017) showed a SNP by trauma exposure interaction on the risk of alcohol misuse. As the multiple testing burden for this kind of design is substantial, large sample sizes are needed to test G×E on a genome-wide level. However, as these samples are becoming increasingly available, the merits of this method might be further explored. Other important characteristics of high quality study methods include using better phenotypical measures, using large discovery and target sample sizes, controlling for covariates and taking into account possible rGE. Authors should report on rGE analysis (that controlled for covariates), and ideally the G×E analysis should control for the effects (for example using structural equation modeling).

Third, future studies should report more completely and transparently on statistics, such as effect size and achieved power level. Also, more attention should be given to null results, so that in future meta-analyses unbiased effect sizes can be estimated (see Fig. 4).

## Strengths and limitations

This is the first review focusing on and comparing multiple polygenic methods for assessing G×E in multiple substance use outcomes. Patterns of results could be compared across different methods, outcomes, and predictors. The quality assessment provided insight in important lacunas in study methodology and gave some suggestion that study quality influences the patterns of results.

The heterogeneity of the included studies introduced some important constraints for the review. No meta-analysis could be attempted and we had to devise our own method to visualize study quality. As we tried to integrate all findings in one comprehensive interpretation, some detail was inevitably lost. Another limitation lies in the fact that some of the studies did not set out to test G×E, but rather included it as a secondary analysis. This might have contributed to the fact that details on methods and results could sometimes be retrieved only with difficulty. Also, it may have biased results, as studies in some cases seemed to test an interaction with a variable that proved to have a main effect, rather than a G×E effect predicted based on the literature.

## Conclusion

The current review summarized literature investigating if environmental and polygenic factors interact in influencing alcohol, tobacco, and cannabis use phenotypes. There are important limitations to the literature, concerning overall study quality, failure to formulate directional hypotheses, inconsistent reports of statistics (effect sizes), and a great lack of replication studies. It is likely that some publication bias exists.

Because of these limitations, it is difficult to draw conclusions about the existence of G×E effects in substance use. Before any substantive claims can be made, it is crucial that some steps are undertaken, such as using more sophisticated methods and direct replication attempts of G×E findings. Although still weak, there is some evidence that polygenic G×E effects are a factor in the etiology of substance use, with PS being the best measure of polygenic risk. Studies suggest that environmental factors can influence the effect of genetic predisposition, either by enlarging its (positive or negative) effects, or by reversing those. Additional work is needed before firm conclusions can be drawn about the importance of G×E in the etiology of substance use.

G×E research has the potential to give crucial insight in biopsychosocial mechanisms underlying substance use that might be leveraged for clinical applications. For example, polygenic scores (Musci et al. 2015a, b) and even single genetic variants in the nicotinic receptor genes (Sarginson et al. 2011) can predict who will respond favorably to smoking interventions. In the future, well-conducted G×E studies have the potential to improve possibilities for clinical applications.

## Electronic supplementary material

Below is the link to the electronic supplementary material.

Supplementary material 1 (DOCX 75 kb)
