# Supplementary material for: Systematic Review of Polygenic Gene–Environment Interaction in Tobacco, Alcohol, and Cannabis Use
Source: Behav Genet. 2019 May 20;49(4):349–65. doi: 10.1007/s10519-019-09958-7 (PMC6554261; doi:10.1007/s10519-019-09958-7)
Supplement: Supplementary file 1 — Supplementary material 1 (DOCX 75 kb) [file 10519_2019_9958_MOESM1_ESM.docx]

**Systematic review of polygenic gene-environment interaction in tobacco, alcohol, and cannabis use**

Joëlle A. Pasman, Karin J.H. Verweij, & Jacqueline M. Vink

**Supplementary information**

Supplementary Table SI. Search term combinations for the systematic literature search…………………..2

Supplementary Table SII. Power (in %) under different assumed effect size and sample size……………..3

Supplementary Table SIII. Reported test statistics for GxE terms in each study...……………………………….4

Supplementary Table SIV. Sample characteristics for all included studies…………………………………………12

Supplementary Table SVa. Quality characteristics for the haplotype studies……………………..………….…15

Supplementary Table SVb. Quality characteristics for the candidate-gene score studies….………………18

Supplementary Table SVc. Quality characteristics for the polygenic score studies………..….………………20

| **Supplementary Table SI.** Search term combinations for the systematic literature search | | | |
| --- | --- | --- | --- |
| **search terms**  **(separated by AND)** | substance use | gene-environment | polygenic risk |
| **related terms**  **(separated by OR)** | substance abuse | genotype environment | genome*wide |
|  | dependen* | GxE | GWA$ |
|  | addict* | gene$ environment | pathway genes |
|  | alcohol | gene$ interaction | genetic candidate system |
|  | ethanol | gene$ interplay | multilocus |
|  | nicotine | gene$ moderat* | multi*locus |
|  | smok* | genotype$ moderat* | gene*score |
|  | cigarette* | genetic interaction | SNP*score |
|  | tobacco | genetic interplay | haplotype* |
|  | cannabis | differential susceptibility | PRS |
|  | marijuana | diathesis*stress |  |
|  | marihuana | nature*nurture inter* |  |
|  | hash | gene inter* |  |

**Supplementary Table SII.** Power (in %) under different assumed effect size and sample size.

|  |  | **R^2^ (%)** | | | | | | | | |
| --- | --- | --- | --- | --- | --- | --- | --- | --- | --- | --- |
|  |  | **0.1** | **0.2** | **0.4** | **0.6** | **0.8** | **1** | **2.5** | **5** | **10** |
| **sample size** | **N=100** | 5.6 | 6.2 | 7.4 | 8.6 | 9.9 | 11.3 | 22.5 | 42.5 | 74.2 |
|  | **N=250** | 6.5 | 8.1 | 11.5 | 15.1 | 19.0 | 23.0 | 52.9 | 85.0 | 99.3 |
|  | **N=500** | 8.0 | 11.5 | 19.1 | 27.2 | 35.6 | 43.7 | 85.3 | 99.3 | >99.9 |
|  | **N=1000** | 11.5 | 19.2 | 35.7 | 51.6 | 65.2 | 75.9 | 99.3 | >99.9 | >99.9 |
|  | **N=2000** | 19.1 | 35.8 | 65.3 | 83.9 | 93.3 | 97.5 | >99.9 | >99.9 | >99.9 |
|  | **N=5000** | 44.0 | 76.1 | 97.5 | 99.8 | >99.9 | >99.9 | >99.9 | >99.9 | >99.9 |
| NB. To establish criteria for study sample size evaluation, power analysis was conducted. Effect sizes of individual genetic variants are commonly found to be between R^2^ = 0.1 to 1% (shaded part of table; So et al., 2011). Studies included in the review tested any number of variants between 2 and thousands, so power was calculated between R^2^=0.1 to 10%. In the analysis, a multiple regression model with at least 3 predictors (2 main and 1 interaction effect) and an alpha level of .05 was assumed. Note that most studies included in the review used more than 3 predictors and will probably have reached slightly lower power levels. | | | | | | | | | | |

| Supplementary Table SIII. Reported test statistics for GxE terms in each study. When *p* was not reported, it was calculated when possible from the test statistics. | | | | |
| --- | --- | --- | --- | --- |
| ID | **year** | **1st author** | **test** | **finding** |
| 1^*^ | 2007 | Berrettini | bupropion treatment x hap on smoking cessation at end of treatment  bupropion treatment x hap on smoking cessation at follow-up | χ^2^(7)=20.6, *p=***.004**  χ^2^(7)=16.1, *p=*.**024** |
| 2^*^ | 2007 | Berrettini | bupropion treatment x hap on smoking cessation at end of treatment  bupropion treatment x hap on smoking cessation at follow-up | χ^2^(7)=22.7, *p=*.**002**  χ^2^(7)=8.88, *p=*.260 |
| 3 | 2009 | Oroszi | naltrexone treatment x hap block 1 on alcohol abstinence  naltrexone treatment x hap block 2 on alcohol abstinence | *p=*.**030**  *p=*.070 |
| 4 | 2012 | Chen | intervention x hap on smoking cessation | χ^2^(2)=8.97, *p=*.**020** |
| 5^+^ | 2013 | Brody | prevention x hap in DRD2 on alcohol use  prevention x hap in ANKK1 block 1 on alcohol use  prevention x hap in GABRG1 block 2 on alcohol use  prevention x hap in GABRA2 on alcohol use | *p<*.**001**  *p=*.**008**  *p<*.**001**  *p=.***001** |
| 6 | 2015 | Tyndale | intervention x hap on smoking abstinence | *NA* |
| 7^%^ | 2006 | Lerer | trauma x haplotype on smoking initiation  trauma x haplotype on nicotine dependence | *NA*  *NA* |
| 8^%^ | 2007 | Segman | trauma x haplotype on nicotine dependence  trauma x haplotype on smoking initiation | χ^2^(1)=6.22, *p=*.**01**  *NA* |
| 9 | 2008 | Ducci | childhood abuse x MAOA-B haplotype on alcoholism  childhood abuse x MAOB-C haplotype on alcoholism | χ^2^(1)=4.47, *p=*.**03**  χ^2^(1)=2.11, *p=*.14 |
| 10 | 2010 | Nelson | childhood sexual abuse x haplotype on alcohol use  childhood sexual abuse x haplotype on alcohol dependence | t(1123)=-2.123, *p<*.**017**  OR=0.42, *p=*.**023** |
| 11 | 2010 | Enoch | trauma x haplotype on substance dependence  trauma x haplotype on alcohol dependence | *NA*  *NA* |
| 12^&^ | 2011 | Kranzler | adverse events x haplotype on alcohol dependence in AA female sample  adverse events x haplotype on alcohol dependence in AA male sample | *p=*.17  *p=*.61 |
| 13^&^ | 2011 | Kranzler | adverse events x haplotype on alcohol dependence in EU female sample  adverse events x haplotype on alcohol dependence in EU male sample | *p=*.38  *p=*.13 |
| 14 | 2013 | Ray | trauma x block 1 haplotype 1 versus 2 on alcoholism^a^  haplotype  trauma  trauma x block 1 haplotype 3 versus 2  haplotype  trauma  trauma x block 2 haplotype1 versus 4 on alcoholism  haplotype  trauma  trauma x block 2 haplotype2 versus 4 on alcoholism  haplotype  trauma  trauma x block 2 haplotype3 versus 4 on alcoholism  haplotype  trauma  trauma x block 2 haplotype5 versus 4 on alcoholism  haplotype  trauma  trauma x block 2 haplotype6 versus 4 on alcoholism  haplotype  trauma  trauma x block 2 haplotype7 versus 4 on alcoholism  haplotype  trauma | *p=***.032**  *p<***.0001**  *p=*.43  *p<*.0001  *p=*.051  *p<*.0001  *p=*.43  *p<*.0001  *p=*.43  *p<*.0001  *p=*.99  *p<*.0001  *p=*.74  *p<*.0001  *p=***.040**  *p<***.0001** |
| 15 | 2015 | Handley | childhood maltreatment x haplotype on marijuana dependence | z=2.00, *p=*.**04** |
| 16 | 2017 | Handley | childhood maltreatment x haplotype on alcohol problems via internalizing  childhood maltreatment x haplotype on alcohol problems via externalizing | *NA*  *NA* |
| 17 | 2012 | McGeary | efficacy of bupropion x score on smoking cessation | *NA* |
| 18^+^ | 2013 | Brody | prevention x score on alcohol use | *p<*.**001** |
| 19 | 2013 | David | bupropion x score on time to first relapse in after smoking cessation  bupropion x score smoking cessation at end of treatment | z=-2.4, *p=*.**016**  z=1.25, *p=*.213 |
| 20 | 2015 | Guo | roommate's drinking x medium vs other score on binge drinking in 1^st^ semester  roommate's drinking x medium vs other score on binge drinking in 2^nd^ semester | *NA*  *NA* |
| 21^$^ | 2015 | Guo | roommate's drinking x medium vs other score on drinking in past 2 weeks  roommate's drinking x medium vs other score on drinking in last year | *NA*  *NA* |
| 22 | 2016 | Bountress | parental knowledge x score on emerging adult substance use disorder, mother report of parental knowledge  parental knowledge x score on emerging adult substance use disorder, adolescent report of parental knowledge peer substance use x score on emerging adult substance use disorder, mother report of parental knowledge  peer substance use x score on emerging adult substance use disorder, adolescent report of parental knowledge | β=-0.46, *p<*.**05**  β=0.54, *p<*.**01**  β=-0.19, *p<*.**05**  β=0.51, *p<*.**01** |
| 23^$^ | 2016 | Stogner | parental rejection on adolescent alcohol use  score on adolescent alcohol use^a^ | z=1.05, *p=*.294  z=0.29, *p=*.772 |
| 24^@^ | 2017 | Pasman | education level x score on moderate polysubstance use sample 1  education level x score on problematic polysubstance use sample 1 | z=0.24, p.81  z=0.19, *p=*.85 |
| 25 | 2017 | Pasman | education level x score on moderate polysubstance use sample 2  education level x score on problematic polysubstance use sample 2 | z=2.23, *p=*.**03**  z=1.90, *p=*.06 |
| 26 | 2017 | Coley | parental drinking x score on alcohol drinking days  friend drinking x score on alcohol drinking days  endogenous life events x score on alcohol drinking days  exogenous life events x score on alcohol drinking days  parental drinking x score on alcohol intoxication  friend drinking x score on alcohol intoxication  endogenous life events x score on alcohol intoxication  exogenous life events x score on alcohol intoxication  parental drinking x score on alcohol use disorder  friend drinking x score on alcohol use disorder  endogenous life events x score on alcohol use disorder  exogenous life events x score on alcohol use disorder | z=0.00, *p=*.951  z=-0.20, *p=*.779  z=-0.17, *p=*.912  z=?, *p=*.414  z=0.50, *p=*.668  z=-0.10, *p=*.946  z=-0.10, *p=*.970  z=0.44, *p=*.655  z=1.54, *p=*.134  z=-0.67, *p=*.480  z=0.36, *p=*.720  z=0.92, *p=*.338 |
| 27^#^ | 2015a | Musci | prevention x PS on age of smoking initiation | t(538)=-1.973, *p=*.**049** |
| 28^#^ | 2016 | Musci | prevention x PS on age of cannabis initiation | t(677)=-3.01, *p=*.**003** |
| 29 | 2012 | Vrieze | age cohort x PS on smoking heaviness  age cohort x PS on alcohol use | *NA*  *NA* |
| 30 | 2013 | Meyers | neighborhood cohesion x PS on smoking heaviness  trauma x PS on smoking heaviness | *p<*.**05**  *p<*.**05** |
| 31^”^ | 2014 | Salvatore | parental knowledge x PS on alcohol problems  peer deviance x PS on alcohol problems | t(1114)=2.27, *p=*.**020**  t(1115)=2.11, *p=*.**040** |
| 32^#^ | 2015b | Musci | environmental risk x PS on smoking frequency environmental risk x PS on cannabis use frequency | OR=1.33, *p=*.**038**  OR=0.10, *p=*.**046** |
| 33^~^ | 2016 | Domingue | birth cohort x PS on smoking initiation  birth cohort x PS on smoking initiation alternative statistical model | *p=*.06  *p=*.05 |
| 34^~^ | 2016 | Schmitz | veteran status x PS on smoking initiation  veteran status x PS on smoking heaviness | *NA*  *NA* |
| 35 | 2017 | Li | friend’s substance use x PS on heavy episodic drinking young age group male  friend’s substance use x PS on heavy episodic drinking young age group female  friend’s substance use x PS on heavy episodic drinking older age group male  friend’s substance use x PS on heavy episodic drinking older age group female | z=0.00, *p=*1.00  z=-0.38, *p=*.70  z=0.63, *p=*.52  z=1.32, *p=*.19 |
| 36^@^ | 2017 | Treur | childhood smoke exposure x PS on smoking heaviness  childhood smoke exposure x PS on smoking initiation | OR=0.97-1.09, CI=0.83-1.26  OR=1.05-1.44, CI=0.76-1.88 |
| 37^@^ | 2018 | Mies | stress x PS on alcohol consumption  life satisfaction x PS on alcohol consumption  stress x PS on alcohol problems  life satisfaction x PS on alcohol problems | *p=*.963  *p=*.406  *p=*.568  *p=*.392 |
| 38 | 2018 | Polimanti | trauma x bipolar disorder PS on alcohol misuse^a^  trauma x major depressive disorder PS on alcohol misuse  trauma x schizophrenia PS on alcohol misuse^a^  trauma x bipolar disorder PS on nicotine dependence^a^  trauma x major depressive disorder PS on nicotine dependence^a^  trauma x schizophrenia PS on nicotine dependence | *NA*  z=-2.29, *p=*.**022**  *NA*  *NA*  *NA*  z=1.45, *p=*.146 |
| 39^“^ | 2018 | Salvatore | romantic relationship status x PS on alcohol intoxication  romantic relationship status x subset of PS on alcohol intoxication | *NA*  *NA* |
| ^*+%&$@#”^ Studies denoted with the same symbol used data from identical or overlapping samples.  In bold *p*-values that were below the traditional two-tailed significance level of *p*<.05  Findings indicated with NA: *p-*value for the interaction term (or for the main effects in the case of reversed interaction) was not reported and could not be calculated  ^a^ These findings followed a reversed pattern; thus, the *p*-values for the main effects are included in the analysis | | | | |

| Supplementary Table SIV. Sample characteristics for all included studies. | | | | | | |
| --- | --- | --- | --- | --- | --- | --- |
| ID | **year** | **1^st^ author** | **population** | **ethnicity** | **% female**  **(cases\|controls)** | **Mean age**  **(cases\|controls)** |
| 1^*^ | 2007 | Berrettini | clinical adult | European American | 54\|55^b^ | 45\|45^b^ |
| 2^*^ | 2007 | Berrettini | clinical adult | African American | 84\|64^b^ | 45\|48^b^ |
| 3 | 2009 | Oroszi | clinical adult | European American | 33\|29^b^ | 45\|45^b^ |
| 4 | 2012 | Chen | clinical adult | European American | 46 (total) | 53 (total) |
| 5^+^ | 2013 | Brody | adolescent, families | African American | 53-56 (total)^b^ | 11-17 (total) |
| 6 | 2015 | Tyndale | adult | European American | 43 (total) | 45 (total) |
| 7^%^ | 2006 | Lerer | college students | Jews (mixed) | 100\|100 | 20-30 (total) |
| 8^%^ | 2007 | Segman | college students | Jews (mixed) | 100\|100 | 24 (total) |
| 9 | 2008 | Ducci | adult | native American (mixed) | 100\|100 | 38 (total) |
| 10 | 2010 | Nelson | ascertained families with twins | European Australian | 78\|46 | 42 (total) |
| 11 | 2010 | Enoch | clinical adult | African American | 0\|0 | 46\|34^b^ |
| 12^&^ | 2011 | Kranzler | ascertained families | African American | 44 (total) | 42 (total) |
| 13^&^ | 2011 | Kranzler | ascertained families | European American | 42 (total) | 38 (total) |
| 14 | 2013 | Ray | clinical adult | European American | 61\|70^b^ | 38\|39 |
| 15 | 2015 | Handley | adolescent | American (mixed) | 44 (total) | 16 (total) |
| 16 | 2017 | Handley | young adult | African American | 53\|? | 20\|? |
| 17 | 2012 | McGeary | clinical adult | mixed American | 16 (total) | 50 (total) |
| 18^+^ | 2013 | Brody | families/ adolescent | African American | 53-56 (total)^b^ | 11-17 (total) |
| 19 | 2013 | David | clinical adult | European American | 52 \| 52^b^ | 45 \| 45^b^ |
| 20 | 2015 | Guo | college student | mixed American | 61^b^ | not reported |
| 21^$^ | 2015 | Guo | high school student | mixed American | 51^b^ | 23^b^ |
| 22 | 2016 | Bountress | ascertained young adult | European American | 53 | 21 |
| 23^$^ | 2016 | Stogner | adolescent | mixed American | 52 | 16 |
| 24^@^ | 2017 | Pasman | families/ adult twins | European Dutch | 63 | 35 |
| 25 | 2017 | Pasman | families/ adolescent | European Dutch | 49 | 33 |
| 26 | 2017 | Coley | adolescent | mixed American | 53^b^ | 28 |
| 27^#^ | 2015a | Musci | adolescent/ young adult | mixed American | 46 (total) | ±6-18 (total) |
| 28^#^ | 2016 | Musci | adolescent/ young adult | African American | 47^b^ | ±6-18 (total) |
| 29 | 2012 | Vrieze | adolescent/ young adult twins | European American | 52 | 20 |
| 30 | 2013 | Meyers | adult | African American | 52 | 48 |
| 31^”^ | 2014 | Salvatore | families/ adolescent twin | European Finnish | 53 | 14 |
| 32^#^ | 2015b | Musci | adolescent/ young adult | mixed American | 46^b^ | ±12-22 |
| 33^~^ | 2016 | Domingue | older adult | European American | 58 | ±56-96 |
| 34^~^ | 2016 | Schmitz | older adult | European American | 0 | ±64-68 (total) |
| 35 | 2017 | Li | ascertained families/ adolescent | European American | 57 | 23 |
| 36^@^ | 2017 | Treur | families/ twin | European Dutch | 67 | ±41 |
| 37^@^ | 2018 | Mies | families/ twin | European Dutch | 65 | 43 |
| 38 | 2018 | Polimanti | adult soldiers | European American | 9 | 23 |
| 39^“^ | 2018 | Salvatore | young adult twins | European Finnish | 54 | 22 |
| ^*+%&$@#”^ Studies denoted with the same symbol used data from identical or overlapping samples. In case-control or RCT designs, percentage female and average age are given separately for cases and controls when reported or calculable. Otherwise, numbers for the total sample and/or ranges are used, as indicated with (total).  ^b^ True numbers and percentages might deviate, as not all individuals were included in the GxE analyses. | | | | | | |

| Table SVa. Quality characteristics for the GxE studies using haplotypes as measure of polygenic risk. | | | | | | | | | | | | | | | | |
| --- | --- | --- | --- | --- | --- | --- | --- | --- | --- | --- | --- | --- | --- | --- | --- | --- |
|  |  |  | **design** | | | **control for** | | | | **polygenic method** | | | | **phenotype method** | |  |
| ID | **year** | **1^st^ author** | **type** | **N (cases)** | **power** | **age** | **sex** | **ethni** | **rGE** | **blocks** | **variants** | **genes** | **rationale risk allele** | **outcome measure** | **env measure** | **qual** |
| 1^*^ | 2007 | Berrettini | RCT | 430 (235)^a^ | yes | desc | stat | homo | NA | 1 | 2 | 1 | debatable | s-Q (1) B | NA | +- |
| 2^*^ | 2007 | Berrettini | RCT | 81 (37)^a^ | yes | desc | stat | homo | NA | 1 | 2 | 1 | debatable | s-Q (1) B | NA | +- |
| 3 | 2009 | Oroszi | RCT | 306 (146)^a^ | no | desc | desc | homo | NA | 2 | 10 | 1 | solid | s-Q (?) v-Q (50) | NA | - |
| 4 | 2012 | Chen | RCT | 1073 (941) | no | stat | stat | homo | NA | 1 | 2 | 3 | solid | *unspecified Q* B | NA | + |
| 5^+^ | 2013 | Brody | long RCT | 963-1,134 (?)^a^ | yes | homo | stat | no | NA | 5 | 13 | 4 | debatable | e-Q (2) | NA | + |
| 6 | 2015 | Tyndale | RCT | 654 (442) | yes | stat | stat | homo | NA | 1 | 2 | 3 | solid | *unspecified Q* B | NA | + |
| 7^%^ | 2006 | Lerer | case control | 390 (242)^a^ | yes | stat | NA | desc | no | 2 | 8 | 2 | debatable | s-Q (?) v-Q (6) | s-Q (?) | - |
| 8^%^ | 2007 | Segman | case control | 390 (242)^a^ | yes | stat | homo | desc | no | 2 | 4 | 1 | debatable | s-Q (?) v-Q (6) | s-Q (?) | +- |
| 9 | 2008 | Ducci | case control | 187 (95)^a^ | no | no | homo | desc | no | 2 | 9 | 2 | debatable | I | I | -- |
| 10 | 2010 | Nelson | case control | 1128 (156) | no | no | stat | homo | no | 1 | 8 | 1 | solid | I | I | - |
| 11 | 2010 | Enoch | case control | 350 (72)^a^ | no | stat | homo | rudim stat | desc | 2 | 7 | 1 | debatable | I | v-Q (28) | - |
| 12^&^ | 2011 | Kranzler | case control | 1869 (634) | no | stat | sep | homo | desc | 1 | 3 | 1 | solid | I | s-Q (3) | - |
| 13^&^ | 2011 | Kranzler | case control | 1,211 (330) | no | stat | sep | homo | desc | 1 | 3 | 1 | solid | I | s-Q (3) | - |
| 14 | 2013 | Ray | case control | 2,533 (1,167)^a^ | no | no | stat | homo | no | 2 | 18 | 1 | debatable | I | I | +- |
| 15 | 2015 | Handley | long case control | 326 (179) | no | stat | desc | rudim stat | no | 1 | 4 | 1 | solid | I | records  classification | - |
| 16 | 2017 | Handley | long case control | 280 (163) | no | stat | desc | stat | desc | 1 | 4 | 1 | solid | s-Q (4) | I | - |
| ^*+%&$@#”^Studies denoted with the same symbol used data from identical or overlapping samples. Sample size for the GxE analysis is given with the cases subsample in brackets for case-control and RCT designs. The ‘power’ column summarizes whether a power analysis was reported. The rGE column denotes NA when gene-environment correlation cannot be an issue because of the study design. The ‘blocks’ column provides the number of LD blocks included in the investigation, with ‘variants’ giving the total number of variants tested in these blocks. The ‘measure’ columns give the type of instrument used to measure phenotype, with the number of items provided in brackets for Qs.  ^a^ True numbers and percentages might deviate, as not all individuals were included in the GxE analyses.  Abbreviations: ethni=ethnicity; rGE=gene-environment correlation; env=environmental; qual=quality; RCT=randomized controlled trial; long=longitudinal; desc=description of differences without statistical control; se*p=*separate analyses for levels of confounder; stat=statistical control; rudim stat= rudimentary statistical control; homo=homogeneous sample; NA=not applicable; Q=questionnaire; I=interview; B=biological measure; v-=externally validated; s-=self-developed; e-=used earlier (but not validated). | | | | | | | | | | | | | | | | |

| Table SVb. Quality characteristics for the GxE studies using aggregates of candidate-genes as measure of polygenic risk. | | | | | | | | | | | | | | | | | | | | |
| --- | --- | --- | --- | --- | --- | --- | --- | --- | --- | --- | --- | --- | --- | --- | --- | --- | --- | --- | --- | --- |
|  | |  |  | | **design** | | | | **control for** | | | | **polygenic method** | | | | **phenotype method** | |  |  |
| ID | **year** | | | **1^st^ author** | | **type** | **N (cases)** | **power** | **age** | **sex** | **ethni** | **rGE** | **gen predictor** | **variants** | **genes** | **rationale risk allele** | **outcome measure** | **env measure** | **qual** |  |
| 17 | 2012 | | | McGeary | | RCT | 90 (?) | no | no | stat | no | NA | sum | 4 | 4 | solid/ debatable | s-Q (?) | NA | -- |  |
| 18^+^ | 2013 | | | Brody | | long RCT | 963-1,134^a^ | yes | homo | stat | homo | NA | sum | 3 | 3 | debatable | e-Q (2) | NA | +- |  |
| 19 | 2013 | | | David | | RCT | 792 (410)^a^ | no | stat | stat | homo | NA | sum | 4 | 4 | solid | *unspecified Q* B | NA | + |  |
| 20 | 2015 | | | Guo | | randomized long | 1,003 | no | stat | stat | stat | NA | propensity levels | 5/10 | 3/6 | debatable | s-Q (2) | s-Q (2) | +- |  |
| 21^$^ | 2015 | | | Guo | | long | 1,612 | no | stat | stat | stat | desc | propensity levels | 5/27 | 3/21 | debatable | s-Q (3) | s-Q (3) | +- |  |
| 22 | 2016 | | | Bountress | | long corr | 254 | no | stat | stat | rudim stat | stat | sum | 7 | 6 | solid | I | e-Q (3) e-Q (6) | +- |  |
| 23^$^ | 2016 | | | Stogner | | corr | 1,495 | no | stat | stat | rudim stat | desc | sum | 5 | 5 | solid | s-Q (1) | e-Q (3-5) | +- |  |
| 24^@^ | 2017 | | | Pasman | | long corr | 2,435 | yes | stat | stat | homo | no | sum | 3/7 | 3/7 | solid | s/e/v-Qs | s-Q (1-3) | +- |  |
| 25 | 2017 | | | Pasman | | long corr | 1,173 | yes | stat | stat | homo | no | sum | 3/4 | 3/4 | solid | s/e/v-Qs | s-Q (1-3) | +- |  |
| 26^$^ | 2017 | | | Coley | | long corr | 11,423 | no | stat | stat | rudim stat | desc | sum | 6 | 6 | solid | s/e-Qs (13) | s-Q (24) | + |  |
| Studies denoted with the same symbol used data from identical or overlapping samples. Sample size for the GxE analysis is given with the cases subsample in brackets for case-control and RCT designs. The ‘power’ column summarizes whether a power analysis was reported. The rGE column denotes NA when gene-environment correlation cannot be an issue because of the study design. The ‘gen predictor’ column gives the type of aggregate of candidate-genes that was used, with sum meaning a sum score of the number of risk alleles. The ‘measure’ columns give the type of instrument used to measure phenotype, with the number of items provided in brackets for questionnaires.  ^a^ True numbers and percentages might deviate, as not all individuals were included in the GxE analyses.  Abbreviations: ethni=ethnicity; rGE=gene-environment correlation; env=environmental; qual=quality; RCT=randomized controlled trial; long=longitudinal; corr=correlational; desc=description of differences without statistical control; stat=statistical control; rudim stat= rudimentary statistical control; homo=homogeneous sample; NA=not applicable; env=environmental exposure; Q=questionnaire; I=interview; B=biological measure; v-=externally validated; s-=self-developed; e-=used earlier (but not validated). | | | | | | | | | | | | | | | | | | | | |

| Table SVc. Quality characteristics for the GxE studies using polygenic risk scores as measure of polygenic risk. | | | | | | | | | | | | | | | | |  | |
| --- | --- | --- | --- | --- | --- | --- | --- | --- | --- | --- | --- | --- | --- | --- | --- | --- | --- | --- |
|  |  |  | **design** | | | **control for** | | | | **polygenic method** | | | | | **phenotype method** | |  |  |
| ID | **year** | **1^st^ author** | **type** | **N (cases)** | **power** | **age** | **sex** | **ethni** | **rGE** | **PRS basis** | **corresp pheno** | **discovery N** | **method** | **variants** | **outcome measure** | **env measure** | **qual** |  |
| 27^#^ | 2015a | Musci | long RCT | 539 (258) | no | NA | stat | rudim stat | NA | 1-3 GWAS | moderate | 1292-4696 | weighted sum | *p<*.01 | s-Q (1) | NA | +- |  |
| 28^#^ | 2016 | Musci | long RCT | 678 (479) | no | NA | stat | rudim stat | NA | 1-3 GWAS | weak | 1292-4696 | weighted sum | *p<*.01 | s-Q (1) | NA | +- |  |
| 29 | 2012 | Vrieze | corr | 3,231 | no | stat | stat | stat | NA | MA of GWAS | strong | 31266 | weighted sum of SNPs in RoI | r^2^>.07 | s-/v-Q (1) | NA | + |  |
| 30 | 2013 | Meyers | corr | 399 | yes | stat | stat | stat | desc | MA of GWAS | strong | 32389 | weighted sum | *p<*5*10^-7 | s-Q (1) | v-classification s-Q (5) s-Q (19) | + |  |
| 31 | 2014 | Salvatore | corr | 1,162 | no | homo | stat | homo | desc | 1 GWAS | strong | 4304 | 2 weighted sums | *p<*.05 *p≤* 0.0001 | I | e-Q (4) s-Q (4) | +- |  |
| 32^#^ | 2015b | Musci | long | 556 | no | homo | stat | stat | desc | 1-3 GWAS | moderate | 1292-4696 | quartiles weighted sum | *p<*.01 | I/ Q (?) | s-Q (2) I | +- |  |
| 33^~^ | 2016 | Domingue | corr | 8,904 | yes | stat | stat | homo | desc | MA of GWAS | strong | 74053 | weighted sum | *p=*1 | s-Q (1) | NA | + |  |
| 34^~^ | 2016 | Schmitz | case control | 631 (198) | yes | stat | homo | stat | stat | MA of GWAS | strong | 74053 | weighted sum | *p=*1 | s-Q (1) s-Q (1) | records ? | + |  |
| 35 | 2017 | Li | long | 241 | no | homo | sep | homo | desc | 1 GWAS in related sample | moderate | 1249 | weighted sum | *p<*.05 | I | I v-Q | - |  |
| 36^@^ | 2017 | Treur | corr | 4,072 | no | stat | stat | stat | desc | MA of GWAS | strong | ~70200 (init) ~34200 (heaviness) | 8 LDpred weighted sums | *p=* 0.0001-1 | s-Q (1) | s-Q (1) | + |  |
| 37 | 2018 | Mies | corr | 6705 | yes | stat | stat | stat | no | MA of GWAS | strong | ~67,000 | 9 LDpred weighted sums | *p=*.0001 – *p=*1 | s-Q (3)  v-Q (10) | s-Q (1)  v-Q (5) | ++ |  |
| 38 | 2018 | Polimanti | case control | 10,732 (8,346) \|  6,132 (4,938) | no | stat | stat | stat | desc for 1 PRS | MA of GWAS | weak | 63,766 (BD)  18,759 (MD)  150,064 (SC) | 7-9 weighted sums | *p=*1e-08 - 0.5 | v-Q (13)  v-Q (5) | v-Q (22) | + |  |
| 39 | 2018 | Salvatore | corr | 1,170 | yes | stat | stat | stat | desc | 1 GWAS | strong | 4,304 | 2 weighted sums, 2 selections of DNase sensitive sites | *p=*0.01 & *p=*0.05 | s-Q (1) | s-Q (1) | + |  |
| Studies denoted with the same symbol used data from identical or overlapping samples. Sample size for the GxE analysis is given with the cases subsample in brackets for case-control and RCT designs. The ‘power’ column summarizes whether a power analysis was reported. The rGE column denotes NA when gene-environment correlation cannot be an issue because of the study design. The ‘PRS basis’ column gives the study type whose summary statistics were used to calculate the PRS. The ‘corresp phen’ indicates how strongly the phenotype is related to the original phenotype for which the summary statistics were calculated. The ‘discovery N’ gives the sample size for the study on which the PRS was based. The ‘measure’ columns give the type of instrument used to measure phenotype, with the number of items provided in brackets for questionnaires.  Abbreviations: ethni=ethnicity; rGE=gene-environment correlation; env=environmental; qual=quality; RCT=randomized controlled trial; long=longitudinal; corr=correlational; desc=description of differences without statistical control; se*p=*separate analyses for levels of confounder; stat=statistical control; rudim stat=rudimentary statistical control; homo=homogeneous sample; NA=not applicable; MA=meta-analysis; GWAS=genomewide association study; init=initiation; Q=questionnaire; I=interview (clinical); B=biological measure; v-=externally validated; s-=self-developed; e-=used earlier (but not validated); BD=bipolar disorder; MD=major depressive disorder; SC=schizophrenia. | | | | | | | | | | | | | | | | | | |
